# Expression of *Streptococcus pneumoniae* Virulence-Related Genes in the Nasopharynx of Healthy Children

**DOI:** 10.1371/journal.pone.0067147

**Published:** 2013-06-18

**Authors:** Fuminori Sakai, Sharmila J. Talekar, Keith P. Klugman, Jorge E. Vidal, for the Investigators Group

**Affiliations:** Hubert Department of Global Health, Rollins School of Public Health, Emory University, Atlanta, Georgia, United States of America; Institut de Pharmacologie et de Biologie Structurale, France

## Abstract

Colonization and persistence in the human nasopharynx are prerequisites for *Streptococcus pneumoniae* disease and carriage acquisition, which normally occurs during early childhood. Animal models and *in vitro* studies (i.e. cell adhesion and cell cytotoxicity assays) have revealed a number of colonization and virulence factors, as well as regulators, implicated in nasopharyngeal colonization and pathogenesis. Expression of genes encoding these factors has never been studied in the human nasopharynx. Therefore, this study analyzed expression of *S. pneumoniae* virulence-related genes in human nasopharyngeal samples. Our experiments first demonstrate that a density of ≥10^4^ CFU/ml of *S. pneumoniae* cells in the nasopharynx provides enough DNA and RNA to amplify the *lyt*A gene by conventional PCR and to detect the *lyt*A message, respectively. A panel of 21 primers that amplified *S. pneumoniae* sequences was designed, and their specificity for *S. pneumoniae* sequences was analyzed *in silico* and validated against 20 related strains inhabitants of the human upper respiratory tract. These primers were utilized in molecular reactions to find out that all samples contained the genes *ply, pav*A, *lyt*C, *lyt*A, *com*D, *cod*Y, and *mgr*A, whereas *nan*A, *nan*B, *psp*A, and *rrg*B were present in ∼91–98% of the samples. Gene expression studies of these 11 targets revealed that *lyt*C, *lyt*A, *pav*A and *com*D were the most highly expressed pneumococcal genes in the nasopharynx whereas the rest showed a moderate to low level of expression. This is the first study to evaluate expression of virulence- and, colonization-related genes in the nasopharynx of healthy children and establishes the foundation for future gene expression studies during human pneumococcal disease.

## Introduction

*Streptococcus pneumoniae* (the pneumococcus) is a Gram positive bacterium that causes severe invasive infection such as pneumonia, septicemia, and meningitis especially in children, the elderly and immunocompromised patients [1]–[3]. It has been estimated that the pneumococcus is responsible for 14.5 million cases of disease worldwide and more than 800,000 deaths in children under five each year [4]. Colonization of the nasopharynx is a necessary step along the path to pneumococcal disease (PD) [5], [6]. Upon entering the nasopharynx, and during its residence there, the pneumococcus shares this anatomical and physiological niche with an array of other bacterial inhabitants [7], [8]. Once carriage is established in the nasopharynx, the pneumococcus can remain asymptomatic or migrate through the Eustachian tubes to cause otitis media, descend down the respiratory tract to cause pneumonia, or invade the bloodstream through the respiratory epithelium to cause bacteremia or meningitis [6], [9]. The mechanism(s) behind this migration, preceding disease, is not fully understood.

The prevalence of pneumococcal carriage increases in the first few years of life, peaking at approximately 50–70% in hosts 2–3 years of age and decreasing thereafter until stabilizing at 5–10% in hosts over 10 years of age in industrialized countries [5], [10]–[14]. Studies have reported carriage rates as high as 60% in adults from some developing countries [12], [15]. Carriage studies have classically utilized bacteriologic cultures, and more recently molecular detection using highly sensitive quantitative PCR (qPCR) reactions. These reactions target selected genes found in most screened *S. pneumoniae* isolates and genome-sequenced strains, (e.g. *lyt*A, *ply* or *cps*4A) [16], [17].

To date, at least 93 capsular serotypes have been identified among *S. pneumoniae* strains [6], [18], [19]. Prevention of PD in children has been achieved by vaccination with pneumococcal conjugate vaccine (PCV), the basis for which is induction of a protective antibody response against the bacterial polysaccharide capsule [6], [20]. Although vaccination has been documented as effective for reducing PD mortality and burden, it seems clear that vaccines with greater coverage, based on proteins (non-capsular antigens) common to all serotypes, will be needed in the future [20].

The ideal protein antigen is one that is present on the cell surface, expressed during nasopharyngeal (NP) carriage and in all stages of the disease (e.g. in lungs during pneumonia) and highly conserved within all serotypes. Animal models of PD and *in vitro* cultures of human respiratory cells have allowed the identification of a number of factors implicated in colonization of the nasopharynx and in pathogenesis [3], [21]. These factors include the capsular polysaccharide, pneumococcal pneumolysin (Ply), adhesins, several proteins implicated in fratricide and regulators. Some of the best characterized candidates as proteinaceous components of new vaccine formulations are Ply [22], [23], pneumococcal surface protein A (PspA) [24], [25], pneumococcal surface protein C (PspC) [26] and pneumococcal surface antigen A (PsaA). There is a lack of information, however, to evaluate expression of these vaccine candidates or other pneumococcal proteins in the human nasopharynx during carriage, or in any other anatomic site during disease.

A few studies have evaluated the presence of some virulence determinants in pneumococcal strains isolated from disease cases or strains isolated form the nasopharynx of healthy children. For example, the gene *psp*A, which encodes PspA (a vaccine candidate), was detected in 99% of carriage strains and invasive strains isolated in Spain [27]. The *rrg*C gene (encoding a protein implicated in biogenesis of the pneumococcal pilus) was amplified by PCR in 21.4% of strains isolated from a Native American population [28] whereas another pilus-associated gene, *rlr*A, was detected in ∼27% of *S. pneumoniae* invasive strains isolated in Portugal [29]. Genes encoding colonization factors such as the neuraminidase genes, *nan*A and *nan*B, were detected in all carriage strains and 96% of invasive isolates, respectively [30]. Our recent studies also demonstrated that all *S. pneumoniae* whether isolated from healthy children or from invasive diseases, encode the gene *lux*S coding for the quorum sensing enzyme LuxS, a regulator of biofilms and nasopharyngeal carriage [31]–[33].

While studies of expression of pneumococcal genes have generally utilized different animal models of carriage and disease [33]–[35], there is virtually no information available to directly measure gene expression in the pneumococcus natural niche, the human nasopharynx. This is in part due to a lack of protocols to isolate high quality pneumococcal RNA from human samples. The potentially low yield of RNA also impairs the use of high-throughput technology for gene expression studies. In this work we developed protocols to analyze pneumococcal RNA that was purified from human NP samples and conducted a study that: 1) identified the bacterial load required to obtain enough pneumococcal nucleic acids for downstream molecular studies, 2) evaluated the prevalence of virulence determinants including genes encoding adhesins, toxins, and regulators implicated in virulence (directly in DNA purified from NP samples collected from healthy children [14]) and 3) investigated the expression of these genes encoding virulence- and colonization-related factors and regulators shown to be important for persistence and virulence.

## Materials and Methods

### Strains

Streptococci (normal flora strains) and other strains utilized in this study were previously characterized [36] and kindly provided by Dr. Lesley McGee from the Centers for Disease Control and Prevention (CDC). Strains included: *S. infantis, S. oralis, S. anguinosus, S. intermedius, S. sobrinus, S. pseudopneumoniae, S. mitis, S. parasanguinis, S. australis, S. mutans, S. peroris, S. oligofermentans, S. intestinalis, S. vestibularis, S. cristatus, S. salivarius, S. gordonii, S. sanguinis, S. sinensis and Dolosigranulum pigrum.* Reference, genome-sequenced, *S. pneumoniae* strain D39 [37] (GenBank accession # NC_008533) and TIGR4 [38] (GenBank accession # NZ_AAGY00000000) were utilized as controls throughout the study.

### Nasopharyngeal samples

The NP samples utilized in this work were part of a study of *S. pneumoniae* colonization conducted in Peru [14]. Children enrolled in the mentioned study were aged 0–3 years of age; more details on the study population can be found in our recent publication [14]. Briefly, samples were collected using rayon swabs and immediately placed in 1 ml of transport medium [skim-milk, tryptone, glucose, and glycerol (STGG) [39] at 4°C and transported to a central laboratory usually within 4 h and then stored at −80°C. The density of *S. pneumoniae* (CFU/ml) in these NP samples had been previously investigated utilizing a molecular approach [14].

### DNA extraction

Strains were grown overnight on blood agar plates, this culture was utilized to prepare a cell suspension in 200 µl of sterile DNA grade water. The suspension was added to 100 µl of TE buffer (10 mM Tris-HCl, 1 mM EDTA, pH 8.0) containing 0.04 g/ml lysozyme and 75 U/ml of mutanolysin and then incubated for 1 h at 37°C in a water bath. To extract DNA from NP samples, 200 µl of STGG were added with the above mentioned buffer and incubated under the same conditions. DNA was extracted using the QIAamp DNA Mini protocol, following recommendations of the manufacturer. DNA was finally eluted in 100 µl, quantified using a NanoDrop spectrophotometer (NanoDrop Technologies, Wilmington, DE) and kept at −80°C until used.

### Conventional PCR and qPCR reactions

Conventional PCR reactions were performed with genomic DNA (∼100 pg) or DNA extracted from NP samples (3 µl) as template, 1 µM of the indicated pair of primers (Table 1), 1× Taq master mix (New England Biolabs) and DNA grade water. All PCR reactions were run in a MyCycler™ Thermal Cycler System (Bio-Rad) under the following conditions: initial denaturing at 95°C for 5 min, followed by 35 cycles of 95°C for 20 s, 55°C for 30 s and 68°C for 1 min, and a final extension at 68°C for 10 min. PCR products were run in 2% agarose gels, stained with ethidium bromide and photographed using a ChemiDoc XRS gel documentation System (Bio-Rad). qPCR reactions were performed with IQ™ SYBR green super mix (BioRad), 300 nM of the indicated primers, and 3 µl of DNA template. Reactions were run in duplicate using a CFX96 Real-Time PCR Detection System (Bio-Rad) and the following conditions; 1 cycle at 55°C for 3 min, 1 cycle at 95°C for 2 min and 40 cycles of 95°C for 15 s, 55°C for 1 min and 72°C for 1 min. Melting curves were generated by a cycle of 95°C for 1 min, 65°C for 1 min and 80 cycles starting at 65°C with 0.5°C increments.

**Table 1:** Primers utilized in this study.

Name	Target	Sequence (5′ to 3′)
JVS1L	*lyt*A	AGTTTAAGCATGATATTGAGAAC
JVS2R		TTCGTTGAAATAGTACCACTTAT
JVS5L	*lux*S	ACATCATCTCCAATTATGATATTC
JVS6R		GACATCTTCCCAAGTAGTAGTTTC
JVS27L	*cps*4A	CGTCTAAGAGTCAGTCTTTCAATA
JVS28R		ATTGATATCCACTCCATAGAGATT
JVS29L	*nana*	CAGTGATAGAAAAAGAAGATGTTG
JVS30R		ATTATTGTAAACTGCCATAGTGAA
JVS31L	*mgr*A	ATCTGTATCGTCAAGAGTTGTTTA
JVS32R		TAAAACCTTTAGTTTAGGCTGATT
JVS35L	16S rRNA	AACCAAGTAACTTTGAAAGAAGAC
JVS36R		AAATTTAGAATCGTGGAATTTTT
JVS53L	*com*D	AACAGTATGAGAGGGATAGAGGAC
JVS54R		GATAAAGGTAGTCCTCGTCAAAAT
JVS55L	*com*C	ATGAAAAACACAGTTAAATTGGAA
JVS56R		TTGTAAAATAAAATCACGGAAGAA
JVS57L	*psp*A	CATAGACTAGAACAAGAGCTCAAA
JVS58R		CTACATTATTGTTTTCTTCAGCAG
JVS59L	*Ply*	TGAGACTAAGGTTACAGCTTACAG
JVS60R		CTAATTTTGACAGAGAGATTACGA
JVS61L	*nan*B	AACTGTCCATATCTCCTATTTTTC
JVS62R		TATTTCTACACCTATCTCACCAGA
JVS63L	*lyt*C	GTCTAGGTTATAGCGGTAAAGAAG
JVS64R		GCTCTTATTTACATATTCCCAGTT
JVS65L	*pav*A	CGATAAAAGCAGTCATAAAATCCT
JVS66R		AGGATTGAGAGATTCTGTACTTGG
JVS67L	*Iga*	CGAATGGCACAAAGATTAAACA
JVS68R		TTCTTCCCTTGAAACTGCTCTC
JVS69L	*rrg*B	CAAAACCACTTGATCCAACAGA
JVS70R		ATTACAAATTCTGCCCCAGCTA
JVS73L	*cbp*A	GCTAATGTAGCGACTTCAGATCAA
JVS74R		AGCTTGGAAGAGTTTCTTCACCTA
JVS75L	*psa*A	CCTGCTGAAAAGAAACTCATTGTA
JVS76R		AGGTCTTGATTTGTTCAGGAGTTC
JVS77L	*psr*P	GCTGCTAGAACTCCAAGTAACACA
JVS78R		TCACAAGTTGGAAATACTTCTGGA
JVS79L	*cod*Y	TATAACGCATAAAATAGCCAAGCA
JVS80R		ATTACATCAATTTTGAAACGCTCA
JVS81L	*cbp*D	TCCTGTTGATTTAGAACCATTTGA
JVS82R		GAGGGAGTGACTTCTTCACAAAAT
JVS83L	*Eno*	GACGGTACTCCTAACAAAGGTAAA
JVS84R		ATAGCTGTAAAGTGGGATTTCAAG
1406F*	*rrn,* intergenic spacer region	TGYACACACCGCCCGT
23Sr*		GGGTTBCCCCATTCRG

* From ref (44).

### RNA extraction, analysis of RNA preparation, and cDNA synthesis

Once thawed, NP samples (200 µl) had 1 volume of RNAprotect Bacteria^®^ (Qiagen) added and were immediately centrifuged for 15 min at 15000×*g* in a refrigerated centrifuge (Eppendorf). Total RNA was then extracted from the pellet using the RNeasy Mini Kit (Qiagen) as outlined by the manufacturer and additionally treated with 2 U of DNaseI (Promega) essentially as previously described [31], [40]. Integrity of our RNA preparations [RNA integrity number (RIN)], and RNA concentration of samples, were obtained by using the RNA 6000 Nano kit or RNA 6000 Pico kit electrophoresis system and the 2100 Bioanalyzer (Agilent technologies). Total RNA (500 pg) was cDNA transcribed using the iScript cDNA synthesis kit (Bio-Rad) and following the manufacturer's instructions.

### Quantitative RT-PCR (qRT-PCR)

Reactions were performed as described above except that in qRT-PCR reactions we utilized 2 µl of cDNA as template. For the relative quantification of mRNA molecules, purified genomic DNA from *S. pneumoniae* reference strain TIGR4 or D39 was serially diluted to prepare standards representing 2.14×10^1^, 4.29×10^1^, 4.29×10^2^, 4.29×10^3^, 4.29×10^4^, or 4.29×10^5^ genome copies. A standard curve was constructed and final copies of each gene target, and therefore mRNA copies, were calculated using the Bio-Rad CFX manager software.

### RT-PCR reactions

Reactions were performed with 5 ng of DNaseI-treated RNA as template. Those reactions contained 500 nM of *lyt*A primers, 1× PCR master mix (New England Biolabs), molecular grade water and 10 units of AMV retrotranscriptase (Promega). Control RT-PCR reactions were similarly performed, except for the omission of reverse transcriptase. Reactions conditions were the following: initial incubation at 42°C for 30 min and denaturation at 95°C for 5 min, followed by 35 cycles of 95°C for 15 s, 55°C for 30 s and 68°C for 1 min and a final extension at 68°C for 10 min. PCR products were run in 2% agarose gels and stained with SYBR® Safe DNA Gel Stain (Invitrogen).

## Results

### Detection of the *lyt*A gene and its transcript in NP samples

**Figure 1:**
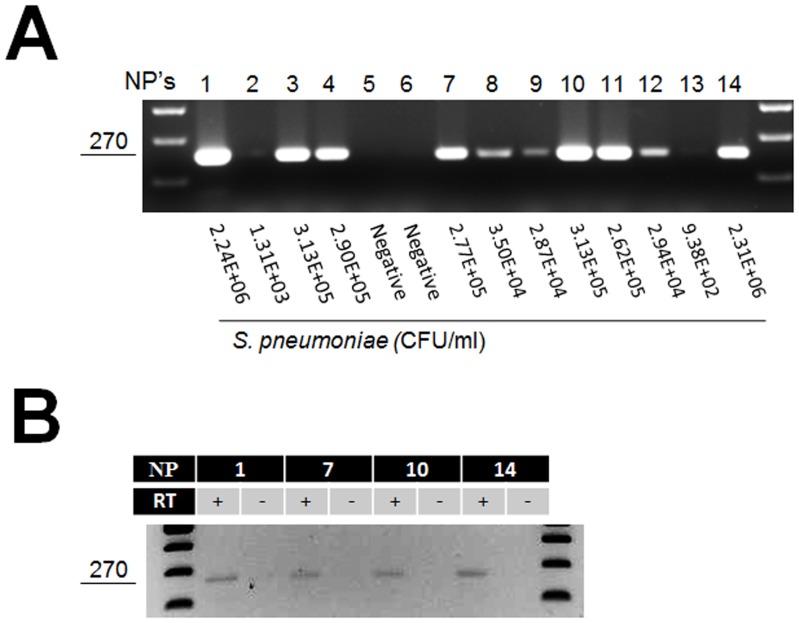
PCR amplification of the *lyt*A gene and RT-PCR detection of its transcript. (A) DNA was extracted from NP samples and utilized as template in PCR reactions amplifying the *lyt*A gene. The loads of pneumococcus cells in each NP sample is indicated below each lane. The larger the bacterial loads, greater the amount of DNA that should be obtained from each sample. This PCR reaction thus detected DNA from NP samples containing at least ∼2.8×10^4^ CFU/ml (samples 8, 9 and 12). (B) RNA was extracted from the indicated NP sample and was utilized as template in RT-PCR reactions targeting the *lyt*A gene. Reactions were added (+) or not (−) with retrotranscriptase (RT). In both panels, the size of the *lyt*A product is shown at left in base pairs.

Since expression of *S. pneumoniae* genes had not been studied in human samples, we first sought to assess whether genes, and expression, could be amplified with conventional PCR and detected by RT-PCR, respectively. To this end, nuclei acids extracted from samples containing different *S. pneumoniae* loads (e.g. 10^2^, 10^3^, 10^4^, 10^5^, or 10^6^ CFU/ml) previously quantified in our laboratory [14], were utilized. As shown in Fig. 1, only those NPs containing ≥10^4^ CFU/ml allowed the PCR amplification of the *lyt*A gene. In contrast, PCR products were absent when the template was DNA from either negative NP samples or from those containing <10^4^ CFU/ml. Ten NP swabs containing each specific *S. pneumoniae* density, or negative samples, were further tested and results were similar to those presented in Fig. 1A. Then, RNA was extracted from NP samples and utilized as a template in RT-PCR reactions that allowed for the detection of a *lyt*A message (Fig. 1B). These results indicated that *S. pneumoniae* DNA or RNA, contained in NP samples with ≥10^4^ CFU/ml, was sufficient to detect by conventional PCR or RT-PCR pneumococcal genes and their transcripts, respectively.

### Development and validation of a panel of primers to amplify *S. pneumoniae* genes

The challenge to detect *S. pneumoniae* genes by PCR, or their transcripts by RT-PCR, in NP samples relies on the potential presence of homologous sequences in species sharing the human upper respiratory tract (including the nasopharynx) with the pneumococcus [41]. Therefore, we first designed and validated, a panel of 21 pair of primers (Table 1) to amplify genes encoding known virulence and colonization factors in the pneumococcus (i.e. *ply*, *nan*A, *iga,* etc.), adhesins (i.e. *pav*A, *pspA, rrg*B, etc.) or genes linked to regulatory functions (i.e. *luxS*, *comC*, *mgr*A and *cod*Y) [1], [9]. These primers were designed based on sequences available from reference *S. pneumoniae* strains D39 and TIGR4 [37], [42].

**Figure 2:**
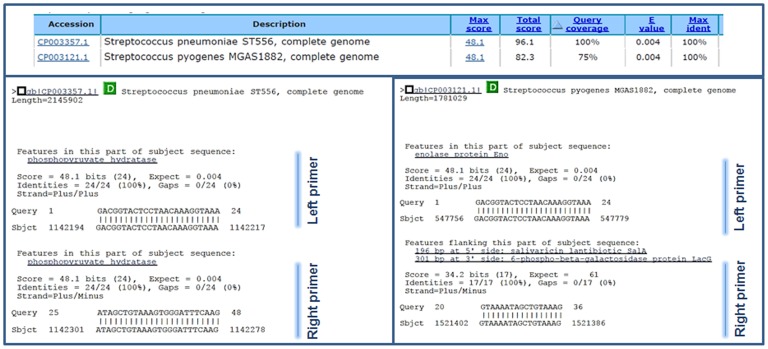
*In silico* analysis of the especificity of primers to amplify the *eno* gene. Sequences of primers designed to ampify the *S. pneumoniae eno* gene were entered into the BLAST website. Among others, total query coverage of 100% and 75% was observed for *S. pneumoniae* strain ST556 or *S. pyogenes* MGAS1882, respectively. Left bottom panel shows that both the left and right primers *in silico* hybridyzed on the *S. pneumoniae eno* gene. Right bottom panel shows hybridization of only the left primer on the *S. pyogenes eno* gene. Part of the righ primer *in silico* hybridized somewhere else in the genome.

Bioinformatic analysis of both forward and reverse primer sequences revealed that, with some exceptions listed below, most of these Table 1 pair of primers would only hybridize *S. pneumoniae* sequences. Besides analyzing each primers individually, both forward (i.e. 22 bp) and reverse sequences (i.e. 22 bp) were also placed together (i.e. 44 bp) and then analyzed again using BLAST. The criteria to define *in silico* hybridization of these concatenated sequences included query coverage >70% and E-score <0.1. Genes/genomes meeting these criteria were individually analyzed to confirm that both primers (i.e. forward and reverse) hybridized within the same gene. For example, Fig. 2 shows that the gene encoding enolase (*eno*) had a perfect match with *S. pneumoniae* strain ST556 (query coverage of 100% and an E-score of 0.004). Both forward and reverse sequences were identified within the same (*eno*) gene (Fig. 2, left bottom panel). Conversely, query coverage of 78% with an E-score of 0.004, which would comply with our criteria for *in silico* hybridization, was detected in *S. pyogenes* strain MGAS1882. However, only 24 bp hybridized in a putative *eno* gene whereas another 17 bp fragment hybridized elsewhere in the genome (Fig. 2, right bottom panel). This *in silico* hybridization was, therefore, not compatible with a potential PCR product.

Overall, our bioinformatics studies revealed that primers amplifying the gene encoding the enolase (*eno*) will also hybridize *in silico* with *S. sanguinis*, *S. parasanguinis*, *S. mitis*, *S. oralis* and *S. gordonii* sequences, whereas primers amplifying *iga* (encoding the IgA protease) will also hybridize with the *iga* gene from *S. oralis*. Genes that were also present in other streptococci included the *psa*A gene found in *S. mitis*, *S. sanguinis* and *S. oralis*, the *cod*Y gene that *in silico* hybridized with *S. pseudopneumoniae* sequences and *cbp*D that hybridize with *S. mitis* and *S. pseudopneumoniae* sequences. These species are found in the healthy human nasopharynx (Table 2) [7], [41].

**Table 2:** PCR amplification of *S. pneumoniae* genes in related species.

	Strain (normal flora of)	Gene target							
		*lytA*	16S rRNA	*eno*	*iga*	*psa*A	*lux*S	*cbp*D	*nan*B
1	*S. infantis* (mouth and pharynx)	−	+	+	−	−	−	−	−
2	*S. oralis* (mouth)	−	+	+	+	−	−	−	−
3	*S. anguinosus* (mouth, pharynx and vagina)	−	+	+	−	−	−	−	−
4	*S. intermedius* (mouth, pharynx and intestines)	−	+	−	−	−	−	−	−
5	*S. sobrinus* (mouth)	−	+	+	−	−	−	−	−
6	*S. pseudopneumoniae* (mouth and pharynx)	−	+	+	−	+	+	+	−
7	*S. mitis* (mouth)	−	+	+	−	+	+	−	−
8	*S. parasanguinis* (throat)	−	+	+	−	−	−	−	−
9	*Dolosigranulum pigrum* (upper respiratory tract)	−	+	−	−	−	−	−	−
10	*S. australis* (mouth)	−	+	+	−	+	+	+	−
11	*S. mutans* (mouth, teeth)	−	+	+	−	−	−	−	−
12	*S. peroris* (teeth and pharynx)	−	+	+	−	+	−	−	−
13	*S. oligofermentans* (mouth)	−	+	+	−	+	−	−	−
14	*S. intestinalis* (intestines)	−	+	+	−	−	−	−	−
15	*S. vestibularis* (mouth)	−	+	−	−	−	−	−	−
16	*S. cristatus* (mouth)	−	+	+	−	−	−	−	−
17	*S. salivarius* (mouth)	−	+	+	+	−	−	−	−
18	*S. gordonii* (teeth, mouth)	−	+	+	−	−	−	−	−
19	*S. sanguinis* (mouth, dental plaque)	−	+	+	−	−	−	−	−
20	*S. sinensis* (mouth)	−	+	+	−	−	−	−	−
21	*S. pneumoniae* (mouth, pharynx, nose)	+	+	+	+	+	+	+	+

**Figure 3:**
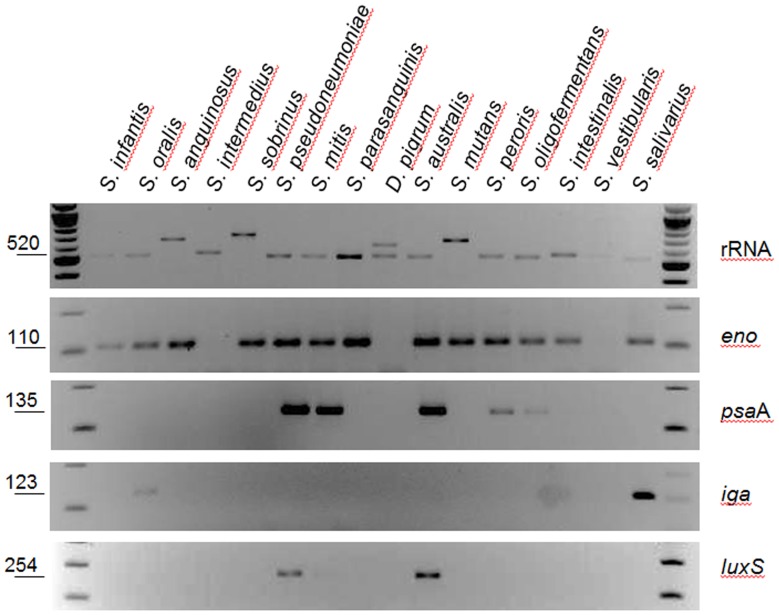
Primers designed to amplify *S. pneumoniae* genes amplify genes in related species. Purified DNA (100 pg) from the indicated species was utilized as template in PCR reactions. Specific genes amplified in those reactions are shown at right of each panel. The size of the expected product, in base pairs, is shown to the left.

To further assess whether these primers would generate a PCR product we performed PCR reactions using DNA templates from a panel of 20 related species that inhabit the human upper respiratory tract [43]. As expected, the 16S rRNA gene was amplified in all species (Table 2). In contrast to bioinformatics studies that only detected hybridization in 5 species, our PCR studies amplified the *eno* gene in most of tested strains whereas the *iga, psa*A, *cbp*D and *lux*S genes were detected in only a few species (Fig. 3 and Table 2). The genes *lyt*A, *lyt*C, *nan*A, *nan*B, *com*C, *com*D, *ply, pspA, psr*P, *codY, rrgB, pavA, cbpA (also known as pspC), cps*4A and *mgrA* were only amplified in control reactions using DNA from *S. pneumoniae* strain D39 or TIGR4 (i.e. some genes are only encoded by either D39 or TIGR4).

Together, these results confirmed that 15 pair of primers were specific for *S. pneumoniae* encoded genes, and therefore could be utilized in reactions containing DNA or RNA purified from NP samples or other anatomic sites.

### Detection of *S. pneumoniae* virulence determinants and regulators in nasopharyngeal samples

Evaluating the presence of genes in a strain isolated from the NP may not represent the whole population of pneumococci found in this anatomic site. This and our unpublished observations that NP samples with high pneumococcal density may contain more than one serotype prompted us to investigate the prevalence of these genes directly in NP samples (N = 50) containing ≥10^6^ CFU/ml of *S. pneumoniae* load and negative samples (N = 22) [14]. DNA extracted from these samples was used as a template in PCR reactions. As expected, almost all reactions that utilized as template DNA from NPs negative to *S. pneumoniae* strains were also negative by PCR for *S. pneumoniae* encoded genes (Table 3). As an internal control that verified the presence of bacterial DNA, and the absence of inhibitors, reactions amplified the bacterial intergenic spacer region from the *rrn* operon [44] utilizing universal primers (not shown).

**Table 3:** Molecular detection of *S. pneumoniae* virulence-related factors and regulators in nasopharyngeal samples.

Target gene	PCR (% positive)		qPCR (% positive)
Virulence and colonization factors	*Sp* * negative NP	*Sp* positive NP	*Sp* positive NP
*csp*4A	0	90.90	97.43
*ply*	0	90.90	100
*rrg*B	0	13.63	92.59
*nan*A	ND**	ND	97.43
*lyt*C	ND	ND	100
*nan*B	0	81	91.89
*psp*A	0	13.63	95.45
*psr*P	0	42.72	45.71
*cbp*A	0	54.54	57.36
*pav*A	0	90.90	100
*lyt*A	0	100	100
Regulators			
*com*D	ND	ND	100
*com*C	0	63.63	71.05
*mgr*A	ND	ND	100
*cod*Y	0	86.36	100

* *Sp: S. pneumoniae.*

** ND: not done.

The most prevalent genes in positive NP samples by PCR were *lyt*A, *pav*A, *ply* and *cps*4A (Table 3). An interesting observation was that the *psp*A gene, which has been described as being encoded by most *S. pneumoniae* strains [27], was only detected by PCR in ∼13% of the *lytA* positive NP samples. We hypothesized that the lesser efficiency of some PCR reactions lowered the limit of detection by conventional PCR. To verify this hypothesis, a more sensitive assay (qPCR) was utilized to reveal that ∼92% of samples were *psp*A positive. Similar findings were obtained when the *rrg*B gene was screened (Table 3). In summary, the *lyt*A, *lyt*C, *pav*A, *ply*, *com*D, *mgr*A and *cod*Y genes were detected in all samples, whereas the prevalence of other gene targets ranged from >90%<99% in *csp*4A, *rrg*B, *psp*A, *nan*A, and *nan*B, to >45%<72% in *psr*P, *cbp*A, and *com*C.

### Gene expression of virulence determinants and regulators in the human nasopharynx

**Figure 4:**
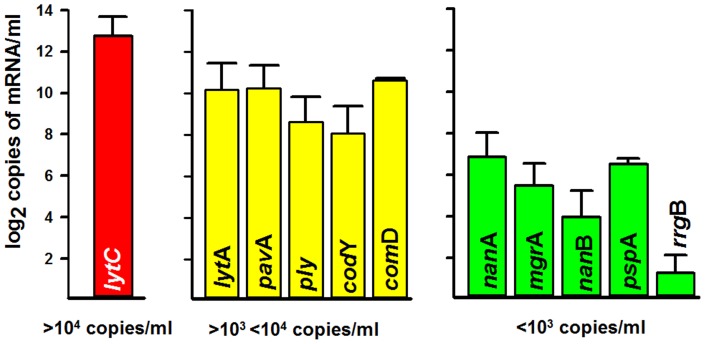
Gene expression studies. RNA was obtained from a set of NP samples; cDNA was generated and utilized in quantitative PCR reactions targeting pneumococcal genes. The graphic was adjusted to show a log2 scale of copies of mRNA/ml of the indicated gene. A red bar represent more than 10^4^ copies/ml, yellow bars correspond to those expressing >10^3^<10^4^ copies/ml whereas green bars represent genes whose transcripts were detected at <10^3^ copies/ml. Error bars represent the standard error of the mean calculated using data from all tested NP samples.

A set of 11 pair of primers, that amplify the most prevalent genes, were chosen to analyze mRNA expression levels in the human nasopharynx. Since not all NP samples contained the target genes, RNA was extracted from 30 samples and its quality and concentration were evaluated using the Agilent RNA 6000 Nano and Pico kits. After synthesizing cDNA, levels of messenger RNA (mRNA) of all screened genes were quantified using a qRT-PCR-based approach. Our studies identified differential gene expression whereby a scale including those showing, in terms of copies/ml of transcript, a low (<10^3^ copies/ml), moderate (>10^3^<10^4^ copies/ml), or high (>10^4^ copies/ml) level of expression could be established. For example, the gene coding for NanA [(*nan*A) a sialidase implicated in colonization [45], [46]] and the adhesin gene *psp*A were detected at low levels of expression (Fig. 4).

The *lyt*A gene, the gene encoding adhesin *pav*A, the pneumolysin gene (*ply*) and the *com*D gene (implicated in competence), showed moderate levels of expression, in comparison with those mentioned above (Fig. 4). In contrast, the lysozyme-encoding gene *lyt*C showed the highest level of expression; most NP samples contained >10^4^ copies/ml of the transcript.

## Discussion

We have analyzed in this study, for the first time, expression of important pneumococcal genes when *S. pneumoniae* is present in the nasopharynx of healthy children. Our results clearly point towards active production of the autolysins LytC and LytA that can be implicated in either fratricide (killing of non-competent pneumococci or other species) or in biofilm formation. We have also demonstrated that transcripts for a vaccine candidate, Ply [22], [47] and an adhesin implicated in carriage and virulence, PavA [48], are detected in pneumoccocal cells residing in the human nasopharynx. We were unable, however, to evaluate mRNA levels of genes encoding other vaccine candidates such as PsaA due to the presence of similar sequences in other species found in the nasopharynx.

Even when we selected NP samples with the highest possible density of pneumococci, the concentration of RNA obtained from our preparations was usually <500 pg/µl. For research purposes, NP swabs collected to detect the pneumococcus are stored in 1 ml of transport medium (STGG) whereby less than 500 ng of bacterial RNA can be obtained with protocols described in this manuscript. As demonstrated, our RNA preparations had enough quality to generate cDNA and analyze expression of a set of selected genes by quantitative RT-PCR. The yield of RNA is, however, an important limitation to the use of bacterial RNA extracted from NP samples for gene expression studies utilizing high-throughput technology (i.e. microarrays, RNA sequencing).

This work provides a panel of primers that were validated against several related strains, most of which are present in the human respiratory tract. Although *in silico* studies were helpful in determining the specificity of primers designed to amplify pneumococcal genes, studies with DNA purified from those 20 related species revealed more nonspecific products which indicates the need for *in vitro* studies to validate *in silico* analyzes. These pneumococcus-specific primers can be utilized to evaluate gene expression in RNA purified from sterile sites (i.e. blood or cerebrospinal fluid) or non-sterile sites (middle ear fluid or bronchoalveolar lavage). For example, a recent study changed our view of the lung microbiome by demonstrating that, although detected with a low density, lungs contain a similar bacterial flora to those of the nasopharynx [49]. Expression of genes in patients experiencing pneumococcal otitis media may also be obtained and compared to those being expressed in the nasopharynx. This information may reveal significant changes in gene expression allowing the pneumococcus to colonize other anatomic sites and to cause disease. These studies are underway in our laboratory.

Studies utilizing conventional PCR and DNA from NP samples to detect pneumococcal genes, correlated with findings utilizing DNA extracted from the strains. For example, the *lyt*A gene could be amplified by PCR in all samples whereas ∼90% of NP samples were positive for the *ply* gene. qPCR studies found, however, more positive samples for most of the target genes. The more striking results where those obtained with the *psp*A gene where our PCR studies detected ∼13% positive samples while quantitative PCR detected *psp*A in >90% of NP samples. This may suggest that a sub-population of pneumococci found in a lower density than that required to be detected by PCR (>10^4^ CFU/ml), but detected by qPCR, could be present in some NP samples. This does not exclude the possibility that in samples, in which results from PCR and qPCR were positive, two populations with both low density and high density encoding the same gene could be present. This hypothesis is also supported by the fact that our NP samples contained a high density of pneumococci (>10^6^ CFU/ml), thereby increasing the chance that those samples would contain more than one serotype or even the same serotype of a genetically different strain.

The gene *lyt*C, which encodes a lysozyme named LytC [50], was detected in 100% of NP samples and was also the gene with the highest level of expression (>10^4^ copies/ml) of the human nasopharynx. Our findings are consistent with a recent observation that a significant increase in anti-LytC antibodies occurs in healthy adult carriers of the pneumococcus in comparison to carriage negative adults [51]. Pneumococcal challenge also induced increased levels of anti-LytC IgG in serum from carriage negative adults [51].

*In vitro* studies have demonstrated that the mature form of LytC is anchored to the cell envelope [50], and it has an optimal enzymatic activity at ∼30°C, which mimics the temperature in the upper respiratory tract [52]. Recent discoveries have revealed that LytC is one of the most important proteins of the pneumococcal biofilm matrix [53]. LytC has also been implicated in fratricide (i.e. lysis of non-competent cells by competent ones) which has been proposed as a mechanism for predation that contributes to virulence by regulating the release of several virulence factors [54], [55]. Overall, our studies and those mentioned above suggest that LytC is important for the pneumococcus to persist in its human host; however whether LytC is also implicated in pneumococcal disease remains to be investigated.

Our studies also demonstrate a low level of expression of the *psp*A gene. Carriage studies in human volunteers inoculated with *S. pneumoniae* strains detected antibodies anti-PspA, indicating that PspA is produced during colonization and/or carriage [51], [56], [57]. This also may indicate that PspA is highly immunogenic since low expression in the human nasopharynx might be enough to stimulate a strong immune response. Another virulence factor that has been associated with antibody production during carriage in children and adults and is an important vaccine candidate is the pneumolysin Ply [51], [58], [59]. Although when purified, Ply may recapitulate lung damage induced by the pneumococcus [60], its role in NP carriage or during biofilm-related otitis media has not yet been fully characterized. Its level of expression in the nasopharynx correlates with a role of Ply in pneumococcal biofilm formation (Shak et al. 2013, unpublished) and production of anti-Ply antibodies in healthy carriers and those experiencing otitis media [58], [59], [61].

In summary, these studies have demonstrated levels of expression of important pneumococcal genes, including vaccine candidates, in the human nasopharynx and have established the basis for future gene expression studies during human pneumococcal disease.
