# Correction: Expression of *Streptococcus pneumoniae* Virulence-Related Genes in the Nasopharynx of Healthy Children

**DOI:** 10.1371/annotation/d0f4e484-1381-42c2-8dbf-015d66a4fdc5

**Published:** 2014-01-03

**Authors:** Fuminori Sakai, Sharmila J. Talekar, Claudio F. Lanata, Carlos G. Grijalva, Keith P. Klugman, Jorge E. Vidal, for the RESPIRA PERU Group

The name of the Investigators group is incorrect. The correct name is the RESPIRA PERU Group.

Dr. Claudio F. Lanata and Dr. Carlos G. Grijalva were not included in the author byline. The new author order and affiliations are as follows:

Fuminori Sakai^1^, Sharmila J. Talekar^1^, Claudio F. Lanata^2^, Carlos G. Grijalva^3^, Keith P. Klugman^1^, Jorge E. Vidal^1^*, for the RESPIRA PERU Group

1 Hubert Department of Global Health, Rollins School of Public Health, Emory University, Atlanta, Georgia, United States of America

2 Instituto de Investigación Nutricional, Lima, Peru

3 Departments of Preventive Medicine, Vanderbilt University School of Medicine, Nashville TN, USA

The correct citation is: Sakai F, Talekar SJ, Lanata CF, Grijalva CG, Klugman KP, Vidal JE, for the RESPIRA PERU Group (2013) Expression of Streptococcus pneumoniae Virulence-Related Genes in the Nasopharynx of Healthy Children. PLoS ONE 8(6): e67147. doi:10.1371/journal.pone.0067147

The were also errors in the acknowledgements section regarding the Investigators Group and it has been updated to reflect the changes to the name. The correct acknowledgements section for the RESPIRA PERU Group is as follows:

RESPIRA PERU Group: Vanderbilt University, Marie R. Griffin, John V. Williams, Kathryn M. Edwards, Philip J. Budge, Yuwei Zhu, Monika Johnson, Carlos G. Grijalva; Emory University, Jorge E. Vidal, Keith P. Klugman; Instituto de Investigacion Nutricional, Ana I. Gil, Hector Verastegui, Claudio F. Lanata.

